# COVID-19 Pandemic Coping, Social Support, and Emotional Health in American Indian and Alaska Native Peoples

**DOI:** 10.1001/jamanetworkopen.2024.46901

**Published:** 2024-11-22

**Authors:** Cole Haskins, Carolyn Noonan, Ann Collier, Richard MacLehose, Dedra Buchwald, Spero M. Manson

**Affiliations:** 1 Department of Behavioral Health Services, Denver Health Medical Center, Denver, Colorado; 2 Department of Psychiatry, University of Colorado, Aurora; 3 Institute for Research and Education to Advance Community Health, Washington State University, Spokane and Seattle; 4 Community & Behavioral Health, Colorado School of Public Health, University of Colorado, Aurora; 5 School of Public Health, University of Minnesota, Minneapolis; 6 Neuroscience Institute, School of Medicine, University of Washington, Seattle; 7 Centers for American Indian and Alaska Native Health, Colorado School of Public Health, University of Colorado, Aurora

## Abstract

**Importance:**

The COVID-19 pandemic has placed a burden on the health of many people, including significant disparities in American Indian and Alaska Native communities.

**Objective:**

This study examines the associations between coping behaviors, social support, and emotional health among American Indian and Alaska Native peoples during the COVID-19 pandemic.

**Design, Setting, and Participants:**

This cross-sectional study included survey data collected from November 2021 to May 2022 from American Indian and Alaska Native adults aged 18 years or older without dementia or other serious cognitive impairments who were seen at 6 urban health organizations primarily in urban settings (in New Mexico, Alaska, Colorado, Minnesota, Utah, and Kansas) in the year prior to the survey.

**Exposures:**

Exposures of interest included avoidant and direct problem-solving coping behaviors and functional and emotional social support.

**Main Outcome and Measures:**

The study outcome was self-reported change in emotional health since COVID-19 pandemic onset. Poisson regression was used to model adjusted multivariate associations. Data were weighted to account for age, nonresponse, and disproportionate representation by clinic population.

**Results:**

A total of 1164 participants were included in the analysis, with a mean (SD) age of 42.5 (13.4) years; 830 (61%, weighted sample percentage) were female. Since COVID-19 pandemic onset, 465 patients (39% weighted) reported worsened emotional health. Problem-solving coping mean (SD) utilization score was 2.5 (0.5), avoidant coping mean (SD) utilization score was 2.3 (0.5), mean (SD) functional social support score was 11.4 (2.9), and 219 participants (18% weighted) reported that emotional support was always available. Using problem-solving coping skills was associated with better emotional health (adjusted prevalence ratio [APR], 0.66 [95% CI, 0.54-0.81] for highest vs lowest tertile), as was always (vs never or rarely) getting emotional support (APR, 0.40; 95% CI, 0.30-0.55) and having more functional support (APR, 0.90 [95% CI, 0.87-0.92] per 1-unit increase in functional social support). In examination of psychological resilience potentially modifying primary exposure associations, no interactions were statistically significant.

**Conclusions and Relevance:**

In this cross-sectional study of urban American Indian and Alaska Native peoples, problem-solving coping skills and more social support were associated with better emotional health during the COVID-19 pandemic. These findings can be used to identify strengths-based approaches to support community emotional health during social upheavals.

## Key Points

**Question:**

Among urban-residing American Indian and Alaska Native peoples, what types of coping behaviors and social support were associated with better emotional health outcomes during the COVID-19 pandemic?

**Findings:**

In this cross-sectional study including 1164 American Indian and Alaska Native participants from 6 states, using problem-solving coping skills and receiving more emotional and functional support were associated with better emotional health.

**Meaning:**

During social upheavals, strengths-based American Indian and Alaska Native community approaches focusing on problem-solving coping and varied social supports may be associated with better emotional health.

## Teaser

This cross-sectional study investigates associations of coping behaviors and social support with emotional health among American Indian and Alaska Native individuals during the COVID-19 pandemic.

## Introduction

The COVID-19 pandemic placed a burden on the physical and emotional well-being of all people, including American Indian and Alaska Native communities. This highlighted protective factors, such as American Indian and Alaska Native community public health achievements, with some of the highest COVID-19 vaccination rates in the US.^1^ American Indian and Alaska Native strengths-based approaches warrant evaluation, as they attenuated the confluence of COVID-19 and emotional health disparities.^2,3,4,5,6,7^ The authors of this study acknowledge further considerations for research partnered with American Indian and Alaska Native peoples, including intergenerational implications of assimilation, boarding schools, cultural suppression, and limited access to health care.^8,9^ Early in the pandemic, elevated COVID-19 incidence, morbidity, and mortality rates were elevated in American Indian and Alaska Native peoples.^10,11^ Prior work revealed worsened emotional health in 46% of American Indian and Alaska Native peoples that was associated with pandemic-related life disruptions and concern for cultural impacts.^12^ Emotional health disparities in American Indian and Alaska Native communities preceded the pandemic, with elevated prior-year and lifetime mental illness diagnoses and suicide rates.^4,5,13^ Despite known COVID-19 and emotional health disparities among American Indian and Alaska Native peoples, there is minimal research on mitigating detrimental outcomes, especially among the majority residing in urban areas.

Coping behaviors may be associated with improved emotional health in the face of pandemic stressors.^14^ The Transactional Theory of Stress and Coping informs the examination of coping, resilience, and well-being during the COVID-19 pandemic.^15,16^ Steps include identifying a stressor, evaluating one’s resources, and adopting coping mechanisms. In problem-solving coping, one directly resolves or reduces a stressor by,^17^ for example, doing something to solve the problem.^18^ Avoidant coping minimizes focus on the stressor without resolution by,^17^ for example, trying to stay away or wishing that things were better.^18^ While neither approach is inherently best, problem-solving coping has been associated with mitigated risk of depression and posttraumatic stress disorder.^19^ To our knowledge, problem-solving and avoidant coping benefits have not been established in American Indian and Alaska Native peoples’ COVID-19 pandemic experiences.^20^

In addition to individual behavior, social connectedness may be associated with improved emotional health.^21,22^ In American Indian and Alaska Native communities, social support is associated with youth resilience^23^ and reduced depression in adults^24,25^ through cultural values.^26^ A prior study of urban American Indian and Alaska Native peoples revealed that COVID-19–related life impacts and concern for cultural impacts were associated with worsened emotional health.^12^ In the present study, these factors were compared with potential protective factors, coping behaviors, and social support.

The aim of this study was to describe coping behaviors and social supports among urban American Indian and Alaska Native peoples during the COVID-19 pandemic and examine associations with emotional health. Drawing on a cross-sectional survey of American Indian and Alaska Native patients seen at 6 large health organizations that serve urban-residing people, this study hypothesized that problem-focused coping and greater social support would be associated with better emotional health during the pandemic.

## Methods

### Study Design

Community Organizations for Natives: COVID-19 Epidemiology, Research, Testing, and Services (CONCERTS) was a cross-sectional study designed to identify and remove barriers to COVID-19 testing among urban-residing American Indian and Alaska Native peoples and to address COVID-19 health disparities; study details are described elsewhere.^12^ In partnership with 6 health organizations serving primarily urban-residing American Indian and Alaska Native peoples, the CONCERTS team constructed cross-sectional surveys to identify barriers, facilitators, and attitudes related to COVID-19 testing and vaccination. These organizations provide a range of services, including health promotion, outreach, medical, behavioral, and dental health care to patients in Albuquerque, New Mexico; Anchorage, Alaska; Denver, Colorado; Minneapolis–St. Paul, Minnesota; Salt Lake City, Utah; and Wichita, Kansas. This study was approved by the Washington State University institutional review board, local review boards of the participating clinics, and the Indian Health Service national institutional review board. This study followed the Strengthening the Reporting of Observational Studies in Epidemiology (STROBE) reporting guideline for cross-sectional studies whenever able. Participants provided written informed consent.

Patients eligible for study inclusion were seen at any of the 6 clinics in the year prior to enrollment and survey completion (November 2021 to May 2022), were at least 18 years of age, were American Indian or Alaska Native, and did not have a diagnosis of dementia or serious cognitive illness. To ensure adequate enrollment of older adults, sampling was stratified by age (18-54 vs ≥55 years). Participant race was concordant between self-reported and clinic-identified American Indian or Alaska Native identity; further racial or ethnic subcategories were not available. For random sampling, each clinic generated a list of eligible patients from their electronic health records. Patients were invited to participate by their clinic; those with an email address were sent an invitation to participate with informed consent documentation and a link to an online REDCap survey, and those without an email address received physical mail. Up to 4 contact attempts were made over 14 days. Patients individually completed the survey via self-report and were compensated $100. There was a minimum goal of 150 patient participants per clinic, and all received a full study description. CONCERTS study size was determined by balancing feasibility of recruitment and maximizing power for a range of possible outcomes in CONCERTS substudies.

### Survey Development and Measures

The survey was designed to collect information about sociodemographic characteristics, health conditions, social determinants of health, access to care, pandemic effects on health and quality of life, attitudes toward COVID-19, and receipt of and barriers to COVID-19 testing and vaccination. Survey questions were developed based on the National Institutes of Health RADx-UP Common Data Elements and PhenX Toolkit, and when necessary, questions were modified for cultural appropriateness or to better reflect study aims. Staff at each clinic provided feedback on the appropriateness, comprehensibility, community priority, and relevance of survey questions to their populations.

The primary outcome was self-reported change in emotional health since the pandemic started in February 2020, described as the same, better, or worse. Consistent with prior CONCERTS work and highlighting the relative positive outcome of maintaining emotional health during the pandemic, groups reporting worsened emotional health were compared with a group of those with the same or better emotional health as reference.^12^ Coping was operationalized in 2 measures, avoidant and problem-solving coping, adapted from the Ayers coping scale subscales,^17,18^ which have been previously used in American Indian and Alaska Native population studies.^27,28^ Mean summary scores for avoidant and problem-solving domains, ranging from 1 to 3 (use of the strategy “never,” “sometimes,” or “most of the time,” respectively), were categorized approximately into tertiles based on the sample distribution. Tertiles were used to allow for nonlinear associations. Functional social support was assessed using the mean response to 5 questions in the Duke–University of North Carolina Functional Social Support Questionnaire–5 (DUFSS-5),^29^ with possible scores ranging from 1 to 15 (higher scores indicate more support). Emotional social support was reported in terms of a 5-point, Likert-type scale of frequency ranging from “never” to “always.” Pandemic-related risk factors for poor emotional health identified from an earlier CONCERTS publication were included comparatively with protective factors.^12^ COVID-19 life disruption was assessed with a count of 8 possible impacts endorsed; quartiles were constructed for groups with 0 to 1, 2 to 3, 4 to 5, or 6 to 8 endorsed impacts. Concern for cultural impacts was assessed using a count of the number of 6 possible impacts endorsed. These questions addressed potential difficulties practicing or losses of Native languages, loss of elders and wisdom keepers, diminished participation in cultural practices, loss of tribal population, stigma, and similarity to historical illnesses. Covariates included age, sex, marital status (married or partnered; divorced, separated, or widowed; or never married), educational attainment, and global self-rated assessment of health (4-point Likert scale: “poor to fair,” “good,” “very good,” or “excellent”). Sex (male, female) was more consistently reported than gender, and a review of responses indicated concordance when both were available. Psychological resilience was assessed as a potential modifier of associations and was constructed from patient-reported agreement on a 5-point scale adapted from the Brief Resilience Scale (higher scores indicate greater resilience).^30^ Based on the sample distribution, tertiles were created to indicate low, moderate, and high resilience (cut points, **≤**3 or ≥4). Cronbach α was calculated to examine reliability of grouped items: problem-solving coping, α = 0.81; avoidant coping, α = 0.73; functional social support, α = 0.90; Brief Resilience Scale, α = 0.85.

### Statistical Analysis

Descriptive statistics were computed, including means, SDs, and frequencies. Poisson regression models were fit to estimate prevalence ratios (PRs) and 95% CIs. Separate Poisson regression models were estimated for the association of the coping and social support measures with emotional health. Two models were fit for each of the 4 exposures; the first examined unadjusted bivariate associations, and the second examined the association of each exposure variable adjusted for all sociodemographic variables, purposefully selected a priori, including sociodemographic and health variables: age, sex, educational attainment, marital status, and self-rated health.

Modification of associations was assessed by fitting the aforementioned adjusted models, including resilience and the product term for the primary exposures and resilience. Poisson models were selected to avoid biased estimates in logistic regression due to the nonrare outcomes.^31^ All analyses incorporated inverse probability weights to account for age-based sampling, nonresponse according to age and sex, and equal weighting for all 6 clinics to prevent disproportionate representation by larger populations. Two-sided *P* ≤ .05 was considered significant. Analyses were conducted with Stata, version 17.0 (StataCorp LLC).^32^

## Results

The patient populations of the included health care organizations ranged from 1269 to 25 043 people seen in 2019. Of 1450 patients in this study, 1164 (80%) had complete data and were included in the analysis, and following inverse probability weighting, the mean (SD) age was 42.5 (13.4) years. A total of 830 patients (61%, weighted sample percentage) were female and 334 (39% weighted) male, 319 (27% weighted) had an educational attainment of college degree or higher, 474 (42% weighted) were married or partnered, and 948 (82% weighted) reported global health as good or better (Table 1). The eTable in Supplement 1 describes characteristics of participants included and excluded from analysis. Most patients (699 [61% weighted]) reported that their emotional health improved or stayed the same since pandemic onset: improved, 108 (9% weighted); same, 591 (52% weighted); and worsened, 465 (39% weighted). Problem-solving coping behaviors were used slightly more frequently than avoidance, with mean (SD) scores of 2.5 (0.5) and 2.3 (0.5), respectively, of a 3.0 maximum score. Overall, 590 patients (50% weighted) reported usually or always having emotional social support, and 219 participants (18% weighted) reported that emotional support was always available. Functional social support was common, with a mean (SD) score of 11.4 (2.9) of 15.0 maximum. When examining previously described pandemic-related risk factors for poor emotional health, concerns about cultural impacts were reported in a mean (SD) of 3.6 (2.0) of 6.0 possible responses. Of 8 possible difficulties experienced due to the pandemic, the mean (SD) reported number was 3.4 (2.1) of 8.0. The mean (SD) Brief Resilience Scale score was 3.5 (0.8) of 5.

**Table 1.** Characteristics of Urban-Residing American Indian and Alaska Native Peoples

	Participants (N = 1164)^a^
**Sociodemographic and health characteristics**	
Age, mean (SD), y	42.5 (13.4)
Sex	
Female	830 (61)
Male	334 (29)
Educational attainment	
Less than high school	53 (5)
High school graduate or GED	236 (22)
Some college	310 (26)
Associate, occupational, technical, or vocational degree	246 (21)
Bachelor degree or higher	319 (27)
Marital status	
Married or partnered	474 (42)
Divorced, separated, or widowed	235 (19)
Never married	455 (39)
Global self-rated health	
Excellent	99 (9)
Very good	347 (31)
Good	502 (42)
Fair or poor	216 (19)
**Exposures**	
Coping score, mean (SD)^b^	
Avoidant actions	
Total	2.3 (0.5)
Try to stay away from the problem	2.2 (0.7)
Try to stay away from things that make you upset	2.4 (0.6)
Avoid people that make you feel bad	2.6 (0.6)
Avoid the problem by going off by yourself	2.1 (0.7)
Problem solving	
Total	2.5 (0.5)
Do something to make things better	2.6 (0.5)
Try to make things better by changing what you did	2.5 (0.6)
Do something to solve the problem	2.6 (0.5)
Do something in order to get something good out of it	2.5 (0.6)
How often do you get the emotional support you need?	
Always	219 (18)
Usually	371 (32)
Sometimes	282 (24)
Rarely	226 (20)
Never	66 (5)
DUFSS-5 functional social support score, mean (SD)	
Total^c^	11.4 (2.9)
I have people who care what happens to me^d^	4.1 (1.1)
I get love and affection^d^	3.8 (1.2)
I get chances to talk to someone I trust about my personal or family problems^d^	3.7 (1.3)
I can get useful advice about important things in life^d^	3.8 (1.2)
I get help when I need transportation^d^	4.1 (1.2)
Pandemic-related risk factors for poor emotional health, mean (SD), No.	
Difficulties experienced due to the pandemic	
All	3.4 (2.1)
Hourly wages reduced or job loss	564 (48)
Finding child care	193 (15)
Getting food	457 (38)
Getting routine medication or accessing health care	520 (44)
Finding transportation	194 (16)
Losing a loved one to COVID-19	469 (42)
Being isolated from others who are important to me	811 (69)
Missing important events	832 (71)
Concerns about cultural impacts of pandemic	
All	3.6 (2.0)
It will be harder to practice or learn our Native language	528 (45)
We will lose our elders and wisdom keepers	847 (72)
It will be harder to participate in our cultural practices	865 (72)
The population of my American Indian and Alaska Native tribe or group will be reduced	770 (67)
I or my American Indian and Alaska Native tribe or group is being stigmatized or marked or seen in a negative way	458 (37)
It reminds me of how our ancestors were wiped out from similar diseases	834 (71)
Brief Resilience Scale score, mean (SD)	
Total^e^	3.5 (0.8)
I tend to bounce back quickly after hard times^f^	3.8 (1.0)
I have a hard time making it through stressful events^e^	2.6 (1.1)
It does not take me long to recover from a stressful event^f^	3.4 (1.1)
It is hard for me to snap back when something bad happens^f^	2.6 (1.1)
I usually come through difficult times with little trouble^f^	3.3 (1.0)
I tend to take a long time to get over setbacks in my life^f^	2.5 (1.1)
**Outcome**	
Overall emotional health since beginning of COVID-19 pandemic in February 2020	
Improved or stayed the same	
Both	699 (61)
Improved	108 (9)
Stayed the same	591 (52)
Gotten worse	465 (39)

Abbreviations: DUFSS-5, Duke–University of North Carolina Functional Social Support Questionnaire–5; GED, General Educational Development.

^a^ Unweighted sample size. Data are presented as number (percentage) of participants unless otherwise indicated. Percentage results were weighted for nonresponse and may not equal 100% due to rounding.

^b^ Possible scores range from 1 to 3 (“never,” “sometimes,” or “most of the time,” respectively).

^c^ Possible score ranges from 1 to 15, with higher scores indicating more support.

^d^ Possible scores range from 1 to 5 (“much less than I would like” to “as much as I would like”).

^e^ Possible score ranges from 1 to 5, where higher scores indicate more resilience.

^f^ Possible scores range from 1 to 5 (“strongly disagree” to “strongly agree”).

### Coping and Social Support

**Figure 1.**
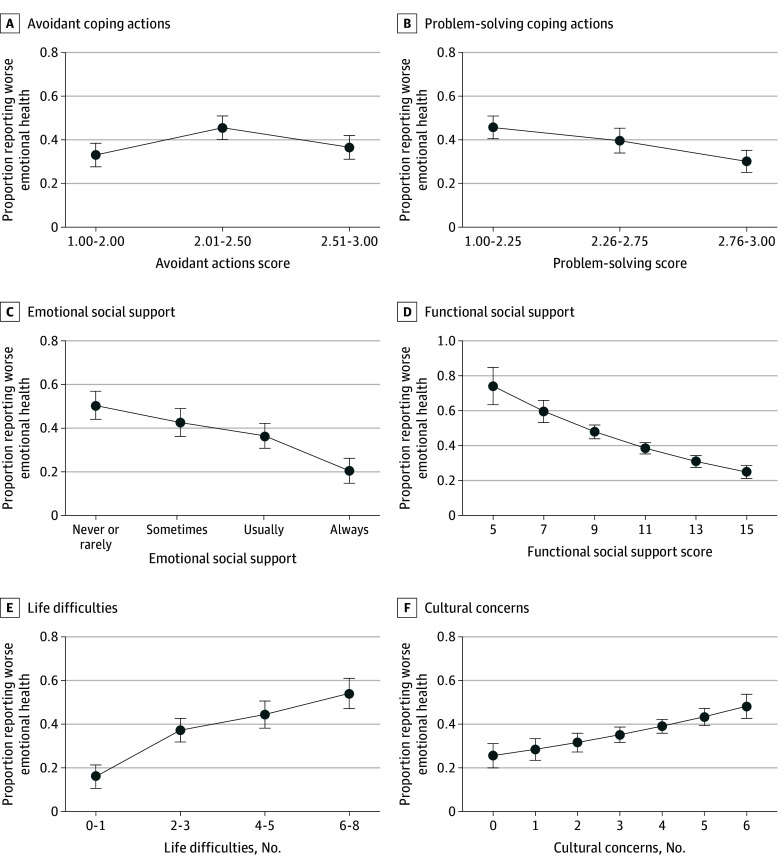
Adjusted Association of Coping Behaviors, Social Support, and Pandemic-Related Difficulties and Concerns With Worse Emotional Health Since the Beginning of the COVID-19 Pandemic Among 1164 Urban-Residing American Indian and Alaska Native Peoples Error bars indicate 95% CIs.

In analyses adjusted for sociodemographic and health covariates, problem-solving coping behaviors were associated with better outcomes or lower prevalence of worsened emotional health (highest tertile: adjusted PR [APR], 0.66 [95% CI, 0.54-0.81], with the lowest tertile as the reference) (Table 2). Avoidance coping behaviors had a nonlinear association in adjusted analysis, with a higher prevalence of worsened emotional health at moderate levels of avoidant actions (middle tertile: APR, 1.38 [95% CI, 1.13-1.68], with the lowest tertile as the reference) (Figure 1). Higher levels of emotional social support were associated with lower prevalence of worsened emotional health (“always”: APR, 0.40 [95% CI, 0.30-0.55], with “never or rarely” as the reference). Higher levels of functional social support were associated with lower prevalence of worsened emotional health (APR, 0.90 [95% CI, 0.87-0.92] per 1-unit increase in functional social support).

**Table 2.** Association of Coping Behaviors, Social Support, and Pandemic-Related Difficulties and Concerns With Worse Emotional Health Since the Beginning of the COVID-19 Pandemic Among Urban American Indian and Alaska Native Peoples, November 2021 to May 2022^a^

	PR (95% CI)	
	Unadjusted^b^	Adjusted^c^
**Sociodemographic and health characteristics**		
Age, y, per 5-y increase	0.95 (0.92-0.98)	0.92 (0.89-0.96)
Sex		
Female	1.25 (1.03-1.51)	1.21 (1.00-1.46)
Male	1 [Reference]	1 [Reference]
Educational attainment		
Bachelor degree or higher	1.30 (1.01-1.69)	1.48 (1.15-1.90)
Associate, occupational, technical, or vocational degree	1.35 (1.04-1.76)	1.39 (1.07-1.80)
Some college	1.22 (0.94-1.58)	1.21 (0.94-1.55)
High school graduate or GED	1 [Reference]	1 [Reference]
Less than high school	1.13 (0.65-1.96)	1.03 (0.59-1.82)
Marital status		
Married or partnered	1 [Reference]	1 [Reference]
Divorced, separated, or widowed	1.09 (0.87-1.36)	1.08 (0.86-1.36)
Never married	1.13 (0.94-1.36)	1.02 (0.85-1.22)
Global self-rated health		
Excellent	1 [Reference]	1 [Reference]
Very good	1.11 (0.75-1.66)	1.12 (0.76-1.64)
Good	1.37 (0.94-2.01)	1.42 (0.99-2.05)
Fair or poor	2.02 (1.37-2.96)	2.22 (1.53-3.21)
**Coping behavior**		
Avoidant actions		
2.51-3.00 (Most use)	1.25 (1.00-1.56)	1.11 (0.89-1.38)
2.01-2.50	1.46 (1.18-1.80)	1.38 (1.13-1.68)
1.00-2.00 (Least use)	1 [Reference]	1 [Reference]
Problem solving		
2.76-3.00 (Most use)	0.62 (0.50-0.76)	0.66 (0.54-0.81)
2.26-2.75	0.82 (0.68-0.99)	0.87 (0.72-1.04)
1.00-2.25 (Least use)	1 [Reference]	1 [Reference]
**Social support**		
Get needed emotional support		
Always	0.38 (0.28-0.52)	0.40 (0.30-0.55)
Usually	0.70 (0.57-0.86)	0.72 (0.59-0.89)
Sometimes	0.83 (0.68-1.01)	0.85 (0.70-1.03)
Never or rarely	1 [Reference]	1 [Reference]
DUFSS-5 functional social support, per 1-unit increase	0.89 (0.87-0.92)	0.90 (0.87-0.92)
**Pandemic-related difficulties and concerns**		
Difficulties experienced due to the pandemic		
6-8	4.05 (2.83-5.80)	3.40 (2.37-4.88)
4-5	3.24 (2.25-4.64)	2.79 (1.93-4.03)
2-3	2.49 (1.72-3.59)	2.34 (1.63-3.38)
0-1	1 [Reference]	1 [Reference]
Concerns about cultural impacts of pandemic per 1-unit increase	1.15 (1.09-1.20)	1.11 (1.06-1.16)

Abbreviations: DUFSS-5, Duke–University of North Carolina Functional Social Support Questionnaire–5; GED, General Educational Development; PR, prevalence ratio.

^a^ Unweighted sample size; results were weighted for nonresponse. Both models include 100% of the analytic sample (N = 1164).

^b^ Includes the indicated variable as the only independent variable, with crude estimates in separate univariate models.

^c^ Includes the indicated variable and all sociodemographic and health variables (age, sex, educational attainment, marital status, and self-rated health) in a single model.

### Pandemic-Related Risk Factors

Previously identified risk factors for poor pandemic emotional health had detrimental associations with mental health in this cohort. Higher prevalence of worsened emotional health was found with more difficulties experienced (most vs least: APR, 3.40; 95% CI, 2.37-4.88) and more concerns about pandemic cultural impacts (APR, 1.11 [95% CI, 1.06-1.16] per 1-unit increase in number of concerns).

### Psychological Resilience Modification Analysis

**Figure 2.**
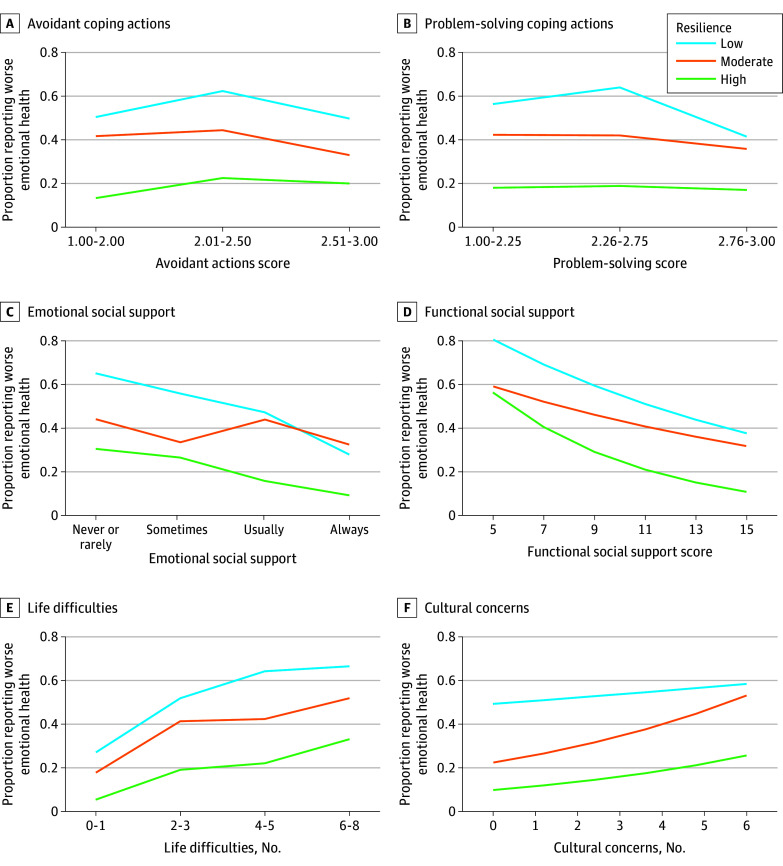
Adjusted Association of Coping Behaviors, Social Support, and Pandemic-Related Difficulties and Concerns With Worse Emotional Health Since the Beginning of the COVID-19 Pandemic, Stratified by Resilience, Among 1164 Urban-Residing American Indian and Alaska Native Peoples

In adjusted models with an interaction term for resilience, no modification of the primary exposure association was revealed (Table 3). As shown in Figure 2, the subgroup with low resilience had the worst rates of emotional health in most categories regardless of primary exposure responses; 230 of the 384 reporting low resilience (60%) also endorsed worsened emotional health (Figure 2, adjusted estimates). Modification of associations by resilience for concerns about cultural impacts of the pandemic was found, with only low resilience not having a significant association with cultural concerns (APR, 1.03; 95% CI, 0.97-1.09) (Table 3).

**Table 3.** Association of Coping Behaviors, Social Support, and Pandemic-Related Difficulties and Concerns With Worse Emotional Health Since the Beginning of the COVID-19 Pandemic, Stratified by Resilience, Among Urban American Indian and Alaska Native Peoples, November 2021 to May 2022^a^

	Adjusted PR (95% CI)			*P* value^b^
	Low resilience (n = 384 [32.5%])	Moderate resilience (n = 432 [37.6%])	High resilience (n = 348 [29.9%])	
**Coping behavior**				
Avoidant actions				
2.51-3.00 (Most use)	0.99 (0.74-1.32)	0.79 (0.55-1.13)	1.50 (0.82-2.74)	.46
2.01-2.50	1.24 (0.95-1.61)	1.07 (0.79-1.43)	1.69 (0.95-2.99)	
1.00-2.00 (Least use)	1 [Reference]	1 [Reference]	1 [Reference]	
Problem solving				
2.76-3.00 (Most use)	0.74 (0.55-0.99)	0.85 (0.61-1.17)	0.94 (0.49-1.80)	.77
2.26-2.75	1.13 (0.91-1.42)	0.99 (0.73-1.35)	1.05 (0.55-1.99)	
1.00-2.25 (Least use)	1 [Reference]	1 [Reference]	1 [Reference]	
**Social support**				
Get needed emotional support				
Always	0.43 (0.25-0.72)	0.74 (0.48-1.12)	0.30 (0.14-0.67)	.10
Usually	0.73 (0.56-0.95)	1.00 (0.72-1.37)	0.52 (0.26-0.98)	
Sometimes	0.86 (0.69-1.07)	0.76 (0.52-1.12)	0.87 (0.46-1.64)	
Never or rarely	1 [Reference]	1 [Reference]	1 [Reference]	
DUFSS-5 Functional Social Support, per 1-unit increase	0.93 (0.90-0.96)	0.94 (0.90-0.98)	0.85 (0.79-0.92)	.07
**Pandemic-related difficulties and concerns**				
Difficulties experienced due to the pandemic				
6-8	2.46 (1.48-4.08)	2.92 (1.72-4.95)	6.15 (1.82-20.8)	.78
4-5	2.37 (1.43-3.95)	2.39 (1.37-4.15)	4.08 (1.29-12.9)	
2-3	1.92 (1.13-3.28)	2.33 (1.38-3.95)	3.55 (1.13-11.1)	
0-1	1 [Reference]	1 [Reference]	1 [Reference]	
Concerns about cultural impacts of the pandemic, per 1-unit increase	1.03 (0.97-1.09)	1.16 (1.07-1.25)	1.17 (1.01-1.37)	.03

Abbreviations: DUFSS-5, Duke–University of North Carolina Functional Social Support Questionnaire–5; PR, prevalence ratio.

^a^ Unweighted sample size; results were weighted for nonresponse. Models were adjusted for age, sex, educational attainment, marital status, and self-rated health. Resilience was defined by the Brief Resilience Scale: cut point for low resilience was scores of 3 or lower and for high was scores of 4 or higher.

^b^ *P* value for interaction.

## Discussion

In this cross-sectional study among urban-residing American Indian and Alaska Native peoples receiving health care at 6 urban health organizations, participants reported using problem-solving coping behaviors more often than avoidance, and only the former had associations with better emotional health. Greater levels of emotional and functional social support were associated with better emotional health. These associations were presented in comparison with known factors of pandemic-related stressors and concern for cultural impacts associated with worsened emotional health during the pandemic.^12^

### Coping Behaviors

Higher utilization of problem-solving but not avoidance coping behaviors was associated with better emotional health. In a different population of American Indian and Alaska Native peoples, problem-focused coping techniques were common during the COVID-19 pandemic.^33^ In a study of US individuals during the early pandemic, emotion regulation skills, problem solving, and distraction coping were associated with lower depression, anxiety, and stress.^34^ Positive reframing and problem-solving coping during the pandemic have been associated with lower levels of depression.^35^ Resilience, adaptive coping, and social support have all been shown as important for mitigating the association of the pandemic with developing acute stress disorder.^36^ Neither avoidant nor problem-solving coping behaviors are inherently good or bad, but they are situation dependent. This study interpreted problem-solving and avoidant coping through the lens of the COVID-19 pandemic; these forms of coping are not mutually exclusive, and flexibility in use and a wider range of coping skills warrant further study.

### Social Support

Emotional and functional social support were associated with better emotional health outcomes. Social support is an important factor in coping with stress, as reaffirmed in context of the COVID-19 pandemic.^36,37^ Although emotional support may directly benefit emotional health, functional support is critical in alleviating daily barriers to care, including obtaining transportation, child care, food, and necessities.^38^ In a study of older American Indian adults, individuals with more social support reported fewer depressive symptoms.^24^ The COVID-19 pandemic has led to social isolation and related distress^39^; this study reaffirms the importance of social support and mobilization of American Indian and Alaska Native community strengths to improve health outcomes.

### Pandemic Stressors

A prior CONCERTS survey of American Indian and Alaska Native peoples spanning January to May 2021 revealed life disruptions during the pandemic, and concern for cultural impacts was associated with worsened emotional health.^12^ After accounting for such measures, having COVID-19 was not associated with changes in emotional health.^12^ Although drawn from the same clinic populations, CONCERTS is not longitudinal, and patients in the 2 surveys differed. Over time, people may have adapted to changes engendered by the pandemic, although these risk factors remain. Problem-solving coping and social support hold promise for mitigating negative associations.

### Resilience

Psychological resilience modification analyses were performed to examine the complex dynamic among coping, social support, and emotional health. Degree of resilience did not modify protective associations of coping or social support. Resilience has been considered as either a personality trait or a dynamic state, with some disagreement in the literature.^40^ The coping survey questions captured dynamic behaviors, whereas the Brief Resilience Scale describes general traits. Resilience in this study may reflect an overall response to adversity, with coping behaviors as actualized efforts; the 2 may function independently. Regardless of coping or social support, 60% of the subgroup with low resilience reported worsened emotional health; those endorsing higher levels of resilience had better emotional health. Similarly, 1 study found that resilience may mitigate pandemic-related associations with posttraumatic stress disorder symptoms.^41^

Resilience training can protect an individual from trauma-related disruptions of emotional health.^42^ Mindfulness and relaxation activities, self-care behaviors, and connecting with social support have been deployed by health care organizations during the pandemic, with beneficial associations.^43^ A study describing psychological first aid highlighted themes for best practices pertinent to health care workers serving American Indian and Alaska Native communities. Examples include strengths-based approaches and language, acknowledgment of the historical influence of pandemics, accommodating differences between urban and rural communities, and promoting connectedness to ameliorate social and cultural isolation.^44^

A statistically significant modification by resilience of the association with concerns about cultural impacts of the pandemic was observed; paradoxically, patients reporting low resilience were the only group with no association between cultural concerns and emotional health. Because resilience is conceptually nuanced and heterogeneous in the literature,^40^ interstudy comparison is challenging. There is a literature gap in the study of pandemic impacts, with minimal examination of culture relevant to American Indian and Alaska Native communities, limiting generalizability. American Indian and Alaska Native community resilience acknowledges a collective nature to resilience, with group strengths and interpersonal relationships influencing individual resilience^45^; this study’s resilience measure did not integrate social domains. Furthermore, subgroup analysis of a categorical Brief Resilience Scale measure introduced smaller sample sizes and the possibility of statistical artifact. Because the subgroup that reported less resilience had a higher prevalence of worse emotional health and resilience did not modify associations with pandemic life disruptions, this subgroup may prioritize immediate life impacts over potential impacts to culture. Nuances of the interface between resilience and pandemic cultural concerns warrant further exploration.

### Limitations

The cross-sectional survey design of this study includes limitations not permitting causal attribution; however, the outcome was phrased to imply temporality to now vs before the COVID-19 pandemic. Emotional health, coping, and social support are dynamic, and the survey represents a snapshot of health. Patient-reported change in emotional health carries significance for communities but is not equivalent to nuanced measures of mental health or a substitute for a clinical examination. Individuals experiencing barriers to care may have been less likely to participate, and those seeking care during or immediately preceding the pandemic may have characteristics dissimilar to those in a different period. Coping behaviors likely have evolved during the pandemic and therefore are best examined in a longitudinal design. Additionally, cross-sectional outcome-exposure associations could have reverse causal directionality. Individuals with worsened emotional health may experience recall bias and view coping behaviors and social support negatively. While the sample size was robust for a study of American Indian and Alaska Native peoples (N = 1164), it may have limited power to detect findings, such as null findings in the modification analysis. Generalizability should be carefully considered given substantial diversity among tribal populations and urban and rural differences. In addition, the response rate in this survey was relatively low, raising the possibility of selection bias.

## Conclusions

In this cross-sectional study of urban-residing American Indian and Alaska Native peoples, problem-solving coping behaviors and more social support were associated with better emotional health during the COVID-19 pandemic. Previously described detrimental associations of emotional health with pandemic life disruptions and concern for cultural impacts persisted,^12^ although the prevalence of worsened emotional health decreased relative to the earlier study. These results highlight the importance of interventions to bolster beneficial coping behaviors and draw on American Indian and Alaska Native community strengths and social support to improve emotional health during the COVID-19 pandemic.

## Notes

Supplement 1. **eTable.** Sociodemographic and Health Characteristics Among Participants Included in the Analysis and Those Excluded Due to Missing Data for Key Analysis Variables

Supplement 2.
Data Sharing Statement
