# Supplementary material for: COVID-19 Pandemic Coping, Social Support, and Emotional Health in American Indian and Alaska Native Peoples
Source: JAMA Netw Open. 2024 Nov 22;7(11):e2446901. doi: 10.1001/jamanetworkopen.2024.46901 (PMC11584921; doi:10.1001/jamanetworkopen.2024.46901)
Supplement: Supplement 1. — eTable. Sociodemographic and Health Characteristics Among Participants Included in the Analysis and Those Excluded Due to Missing Data for Key Analysis Variables [file jamanetwopen-e2446901-s001.pdf]

Supplementary Online Content

Haskins C, Noonan C, Collier A, MacLehose R, Buchwald D, Manson SM. COVID-19 pandemic coping, social support, and emotional health in American Indian and Alaska Native peoples. *JAMA Netw Open*. 2024;7(11):e2446901.
doi:10.1001/jamanetworkopen.2024.46901

eTable. Sociodemographic and Health Characteristics Among Participants Included in the Analysis and Those Excluded Due to Missing Data for Key Analysis Variables

This supplementary material has been provided by the authors to give readers additional information about their work.

© 2024 Haskins C et al. *JAMA Network Open*.

eTable: Sociodemographic and health characteristics among participants included in the analysis and those excluded due to missing data for key analysis variables

	Included	Excluded
	**N[1] (%)**	**N[1] (%)**
Age in years, mean (SD)	42.5 (13.4)	46.0 (14.2)
Female	830 (61%)	204 (63%)
Education		
College degree or higher	53 (5%)	14 (7%)
Associate, occupational, technical, or vocational degree	236 (22%)	61 (21%)
Some college	310 (26%)	73 (27%)
High school graduate or GED	246 (21%)	58 (21%)
Less than high school	319 (27%)	72 (25%)
Marital status		
Married or member of an unmarried couple	474 (42%)	111 (38%)
Divorced, separated, widowed	235 (19%)	84 (31%)
Never married	455 (39%)	84 (30%)
Global self-rated health		
Excellent	99 (9%)	32 (12%)
Very good	347 (31%)	76 (26%)
Good	502 (42%)	121 (41%)
Fair/Poor	216 (19%)	53 (21%)

[1] Unweighted sample size, percentage results weighted for non-response and may not equal 100% due to rounding; N=0 missing age, N=3 missing sex, N=8 missing education, N=7 missing marital status, N=4 missing self-rated health

© 2024 Haskins C et al. *JAMA Network Open*.
