# Supplementary material for: COVID-19 Pandemic Coping, Social Support, and Emotional Health in American Indian and Alaska Native Peoples
Source: JAMA Netw Open. 2024 Nov 22;7(11):e2446901. doi: 10.1001/jamanetworkopen.2024.46901 (PMC11584921; doi:10.1001/jamanetworkopen.2024.46901)
Supplement: Supplement 2. — Data Sharing Statement [file jamanetwopen-e2446901-s002.pdf]

Data Sharing Statement

Haskins. COVID-19 Pandemic Coping, Social Support, and Emotional Health in American Indian and Alaska Native Peoples. *JAMA Netw Open*. Published November 22, 2024. doi:10.1001/jamanetworkopen.2024.46901

Data
Data available: No

Additional Information
Explanation for why data not available: Data are restricted and available if community partners allow (American Indian and Alaska Native health organizations)
